# Albumin displacement at the air–water interface by Tween (Polysorbate) surfactants

**DOI:** 10.1007/s00249-020-01459-4

**Published:** 2020-09-11

**Authors:** Martin Rabe, Andreas Kerth, Alfred Blume, Patrick Garidel

**Affiliations:** 1 Institute of Chemistry, Physical Chemistry, Martin-Luther-University Halle-Wittenberg, von-Danckelmann-Platz 4, 06120 Halle/Saale, Germany; 2 Present Address: Max-Planck-Institut für Eisenforschung GmbH, Max-Planck-Str. 1, 40237 Düsseldorf, Germany; 3 Boehringer Ingelheim Pharma GmbH and Co. KG, Innovation Unit, PDB, 88397 Biberach an der Riss, Germany

**Keywords:** Protein, Albumin, Tween (polysorbate), IRRAS, BAM, Protein-surfactant interaction

## Abstract

Tween (polysorbate) 20 and 80 are surfactants used for the development of parenteral protein drugs, due to their beneficial safety profile and stabilisation properties. To elucidate the mechanism by which Tween 20 and 80 stabilise proteins in aqueous solutions, either by a “direct” protein to surfactant interaction and/or by an interaction with the protein film at the air–water interface, we used spectroscopic (Infrared Reflection Absorption Spectroscopy, IRRAS) and microscopic techniques (Brewster Angle Microscopy, BAM) in combination with surface pressure measurements. To this end, the impact of both types of Tweens with regard to the displacement of the protein from the air–water interface was studied. As a model protein, human serum albumin (HSA) was used. The results for the displacement of the adsorbed HSA films by Tweens 20 and 80 can partially be understood on the basis of an orogenic displacement mechanism, which depends on the critical surface pressure of the adsorbed protein film. With increasing concentration of Tween in the sub-phase, BAM images showed the formation of different domain morphologies. IRRA-spectra supported the finding that at high protein concentration in the sub-phase, the protein film could not be completely displaced by the surfactants. Comparing the impact of both surfactants, we found that Tween 20 adsorbed faster to the protein film than Tween 80. The adsorption kinetics of both Tweens and the speed of protein displacement increased with rising surfactant concentration. Tween 80 reached significant lower surface pressures than Tween 20, which led to an incomplete displacement of the observed HSA film.

**Electronic supplementary material:**

The online version of this article (10.1007/s00249-020-01459-4) contains supplementary material, which is available to authorized users.

## Introduction

Tweens are non-ionic surfactants which are widely used as co-solutes in various industrial applications, for example in the food industry as emulsifiers, or in pharmaceutical science to stabilise proteins. Especially Tween 20 and 80 are commonly used as co-solutes of therapeutic drugs due to the good tolerance of these two surfactants as parenteral excipients (Gervasi et al. 2018; Dwivedi et al. 2018; Falconer 2019). A number of studies have shown that both Tweens improve the colloidal stability of proteins in an aqueous environment and avoid the formation of protein particles (Carpenter and Manning 2002; Garidel et al. 2015). The presence of particles in biologics is of great safety concern and a lot of effort has focused on monitoring and avoiding it (Den Engelsman et al. 2011; Garidel and Kebbel 2010). Especially, at high protein concentrations, above 1 mM, avoiding the formation of protein particles is challenging (Wang and Roberts 2010; Garidel et al. 2017). In particular, surfactants such as Tween 20 (polysorbate 20) and Tween 80 (polysorbate 80) are used to protect proteins against interfacial stress (Khan et al. 2015). Different stabilisation mechanisms were proposed and discussed, such as (1) competitive adsorption to the hydrophobic interfaces and/or (2) a direct binding to the protein and “covering” of hydrophobic protein patches and/or (3) the formation of protein-surfactant micelle complexes. However, up to now, the actual stabilization mechanism is unclear.

**Fig. 1:**
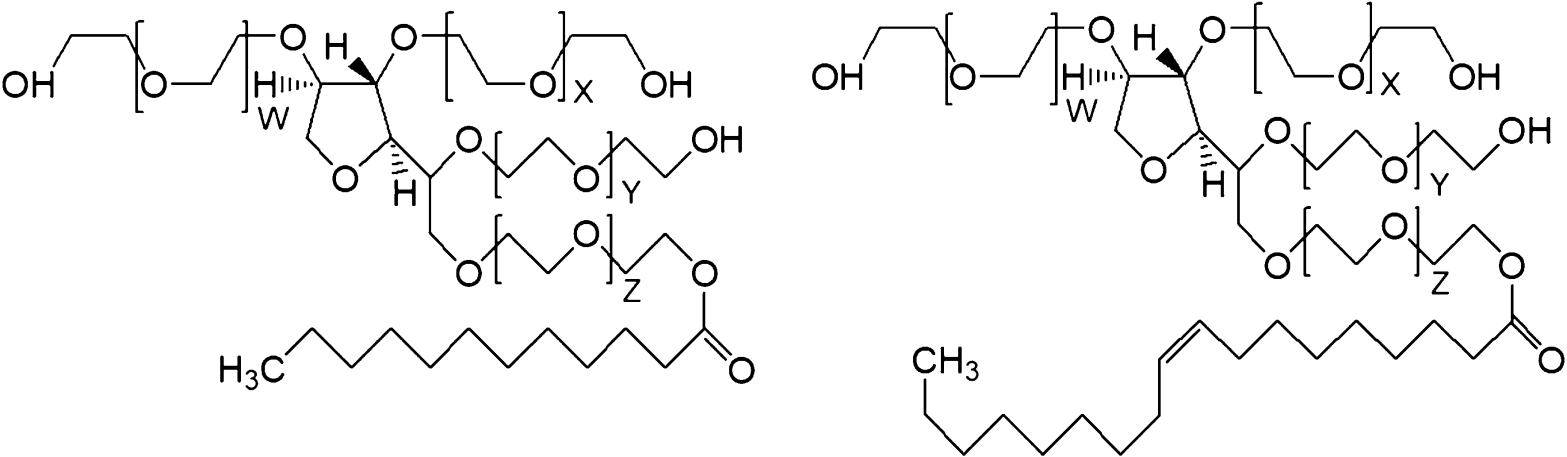
Chemical structures of surfactants Tween 20 (Polysorbate 20) (left) and Tween 80 (Polysorbate 80) (right), with *W* + *X* + *Y* + *Z* = 16–22

Chemically, the Tweens are polyoxyethylene-1,4-sorbitan-monoesters containing a mixture of fatty acids (Dwivedi et al. 2018, 2020). The used and commercially available Tween 20 and 80 are, however, a mixture of molecules related to the parent molecule sorbitan polyoxyethylene fatty acid ester (Fig. 1). According to the EU specifications, Tween 20 is composed of approx. 40–60% of lauric and to approx. 14–25% of myristic acid, whereas Tween 80 is composed of  ≥ 58% oleic acid (Khan et al. 2015). In addition, substantial amounts of polyoxyethylene, sorbitan polyoxyethylene, and isosorbide polyoxyethylene fatty acid mono, di-, tri- and tetra-esters are present (Dwivedi et al. 2020).

Surfactants are added to increase colloidal protein stability in solution as well as at interfaces. Which of these two mechanisms dominate, namely (1) competitive adsorption to hydrophobic interfaces and/or (2) a direct binding to the protein, is not yet understood. Certainly, it may depend on the colloidal and surface-active properties of the protein (Koepf et al. 2018a, b). Tweens, for example, can interact directly with the protein, and especially with exposed hydrophobic protein surfaces, which leads to an improvement of protein solubility and stability, or they may interact with other excipients present in the formulation (Torosantucci et al. 2018). On the other hand, Tweens may compete with proteins for adsorption at the air–water interface, thus hindering proteins from adsorbing to hydrophobic interfaces.

In previous studies, we applied different calorimetric techniques to study Tween–protein interactions. It was found that for human serum albumin, Tween binding was low with binding constants in the range of approx. 10^3^ M^−1^, with 1–3 surfactant molecules binding to the albumin (Garidel et al. 2009). Binding of Tween to another class of proteins, namely to immunoglobulins was also found to be quite weak and negligible, showing that a direct and strong Tween binding to the protein is not the main reason for the colloidal stabilisation effect of immunoglobulins in solution in the presence of Tween 20 or 80 (Hoffmann et al. 2009). These results were supported by McAuley et al. (2009) who investigated by isothermal titration calorimetry the interaction between Tween and lactate dehydrogenase (LDH). Their observation was that no significant interaction between Tween 20 and LDH was found by means of isothermal titration calorimetry. However, these observations were made for bulk solutions and thus did not exclude a competitive interaction of Tween and protein with the air–water interface. Therefore, the question remained open whether the observed stabilization effect of Tween on protein solutions was due to modulations of these interactions at the air–water interface. For an approach to this question a thorough understanding of the properties of adsorption layers forming on protein and surfactant solutions and the mutual interactions of these components on the air-water interface is needed.

The current investigation aims at understanding the displacement kinetics of an albumin film formed at the air–water interface, induced by two surfactants. The air–water interface represents a special interphase with several specific properties (Medders and Paesani 2016). Among these properties, to name the most important, are the geometric nature of the interphase, namely a molecularly flat surface with a boundary region between two phases (air and water) with different dielectric constants. The air side of the air–water interface is an extremely hydrophobic surface (van Oss et al. 2005; Ariga and Hill 2011). This interphase promotes dynamic interactions within the air–water interface, and thus is an interaction region between hydrophobic and hydrophilic molecules. As a consequence of these physical properties of the air–water interface, hydrophobic molecules as well as the hydrophobic side of amphiphilic molecules, such as phospholipids, are attracted and adopt a specific orientation at the interface, with the hydrophobic part of the molecules oriented towards the air phase, whereas the hydrophilic side of the molecule points to the water phase (Garidel and Blume 2005). The primary structure of proteins, i.e. the presence of hydrophobic and hydrophilic amino acid residues provides them with amphiphilic properties such as surface activity (Fernández 2016). In aqueous solutions, the contact area between solvent and hydrophobic residues is minimized. However, on the hydrophobic/hydrophilic air–water interface further reorientation and structural changes may occur. “Soft” proteins may show irreversible changes in the secondary and tertiary structures maximizing the contact area between hydrophobic molecule patches and hydrophobic air while “harder” proteins can resist such changes (Tripp et al. 1995). Furthermore, interface-triggered unfolding may lead to the formation of novel intermolecular interactions which results in the formation of heterogeneous networks at the interface (Morris and Gunning 2008, Koepf et al. 2017, 2018c). As a consequence of the complexity of this process, the adsorption kinetics of specific proteins may differ drastically, especially compared to surfactants. Human serum albumin, used in this study, shows significant surface activity: however, experimental data suggest that no major unfolding occurs upon adsorption (Lu et al. 1999; Makievski et al. 1998). The displacement of protein films formed from common food proteins from the air–water interface by surfactants has been studied thoroughly. These studies resulted in the formulation of an ‘orogenic displacement’ mechanism (Mackie et al. 1999, 2001; Morris and Gunning 2008; Woodward et al. 2009). This mechanism involves that surfactant molecules adsorb into defects of the protein network leading to increasing surface pressure and a stress, which leads to desorption of protein molecules into the sub-phase. Increasing surface pressure leads to folding and buckling of the protein film accompanied by an increase in the protein film thickness and protein material being progressively forced into the sub-phase. Finally, at a distinct critical surface pressure, the protein network collapses and is completely displaced from the interface. During this process, re-adsorption of the protein is inhibited due to the adsorbed surfactant layer.

In this study, we investigate whether this orogenic displacement is a generic mechanism beyond the application to food proteins, i.e. is also valid for parenteral protein drug formulations containing Tweens, as in our former studies human serum albumin (HSA) was used as a model protein (Garidel et al. 2009; Hoffmann et al. 2009). To this end, we first prepared pure albumin films and then injected Tween solutions into the sub-phase underneath the protein film and observed the changes in surface pressure as a function of time. In addition, the spectroscopic technique infrared reflection absorption spectroscopy (IRRAS) was used (Kerth et al. 2004, 2009; Blume and Kerth 2013) allowing us to directly monitor the adsorption processes at the air–water interface by following the intensity of characteristic infrared bands of the protein and the surfactant as a function of time. Brewster angle microscopy (BAM) was applied to gain additional insight into the macroscopic behaviour of the protein film after addition of surfactant into the sub-phase underneath the film (Amado et al. 2008).

## Materials and methods

### Materials

All experiments were performed using sodium citrate buffer (25 mM, pH 6) with sodium chloride (115 mM) at 20 °C. All solutions were prepared and all cleaning procedures were performed with Milli-Q water with a conductivity < 0.055 µS cm^−1^ and a TOC (total organic content) value < 8 ppm. Human serum albumin (HSA; essentially fatty acid free) was purchased from Sigma-Aldrich GmbH (Germany). Protein solutions were filtrated immediately before use through a cellulose acetate syringe filter (pore size: 0.2 µm) and diluted if necessary. Tween 20 (Polysorbate 20) and Tween 80 (Polysorbate 80) (both HP grade) were obtained from Croda International Plc. (UK), sodium chloride and sodium citrate from Sigma-Aldrich GmbH (Germany).

### Brewster angle microscopy (BAM)

Brewster angle microscopy (BAM) experiments were performed with a “MiniBAM” from “Nanofilm Technologies GmbH” (Göttingen, Germany) in combination with a PC equipped with the image capture and editing software “Image Pro Plus 4.0” from “Media Cybernetics” (Silversprings, MD, USA). The image scale was calibrated with a 0.5 mm grid to ± 0.1 mm. The BAM was placed over a Langmuir trough of 87 × 25 × 5 mm^3^, equipped with a magnetic stirrer. Surface pressure was measured using a filter paper as Wilhelmy plate. A dark glass plate with inclined surface was placed under the incident light beam to prevent light reflectance from the trough bottom. The whole apparatus was placed in a darkened box with water reservoirs for the reduction of evaporation. The temperature of the Langmuir trough was kept constant to 20 °C using a circulating water bath (Thermostat F3, Haake, Karlsruhe, Germany).

Prior to every experiment the microscope was adjusted for optimal image quality by means of a clean water interface and the balance was calibrated. For the experiments, citrate buffer (25 mM, pH 6), containing 115 mM NaCl, was used as sub-phase. For measuring protein adsorption in a Langmuir trough different procedures can be applied. Here, a variation of a surface-sweeping technique (Graham and Phillips 1979) was used. First, protein solutions were filled into the Langmuir trough and allowed to equilibrate for ca. 10 min until constant temperature was reached. Next, initially surface-adsorbed protein was carefully suctioned off from the whole surface area using a bevelled plastic tip, followed by fine adjustment of the filling level by suctioning off excess solution with a syringe needle, fixed in height with respect to the trough surface. Directly after the intended filling level was reached, adsorption of new protein from the sub-phase was observed and monitored by surface pressure increase. The protein adsorption phase was considered finished, when the change of the surface pressure had decreased to Δ*π* ≤ 0.2 mN m^−1^ h^−1^, which was usually the case after 2–4 h. Then, stirring of the sub-phase with a magnetic stirrer and without visible eddies or swirls was started. The surface pressure and morphology of the film were monitored for at least 5 more minutes to ensure that no changes were caused by stirring the sub-phase. Next, surfactant injections were performed by piercing through the adsorbed protein layer using a syringe with a thin needle, while stirring the sub-phase. The syringe was held at a shallow angle so that the needle could be carefully placed to the trough bottom to ensure that the injection was performed at the lowest point in the vicinity of the stirrer. For the formation of spread albumin films, an HSA solution (2 mg ml^−1^, in citrate buffer 25 mM, 115 mM NaCl, pH 6) was applied dropwise from a microliter syringe onto a buffer interface until a surface concentration of 1.13 mg m^−2^ was reached, resulting in a surface pressure of ca. 1 mN m^−1^. After an equilibration time of 10 min, the film was compressed to a surface pressure of 17.7 mN m^−1^.

Two different kinds of experiments were performed:

- Multiple injections of aliquots of a surfactant solution into the sub-phase for the investigation of correlations between surfactant concentration, surface pressure and displacement progress.
- Single injection of surfactant for investigation of correlations between surface pressure, displacement progress and elapsed time after injection.

The surface coverage of protein was quantified by analysis of the histograms of the BAM images. Image sections with even illumination, good sharpness, and as few as possible (not more than two) protein islands, and at least 3000 pixels were selected manually. The corresponding histograms were fitted with two Gaussian functions to obtain the ratio of bright to dark area. The values 100% and 0% protein surface coverage were determined manually, whereby 100% was set at the moment of injection and 0% at the moment where no further small protein domains were recognisable. A scheme illustrating the analysis method is given in the SI (Figure S1).

It is important to note that the purpose of this analysis is to quantify protein surface coverage in the form of domains in the µm-range and coherent protein islands are excluded from it. Thus, by definition, a relative protein surface coverage of 0% means there are no “small” (in dimensions of µm) protein domains visible on the surface, but bigger solid protein island (in dimensions of 100 µm) can still be present at the surface. The feasibility of this analysis and thus the data point density depends strongly on the image quality.

### Infrared reflection absorption spectroscopy (IRRAS)

IRRAS measurements were performed in a setup equipped with two Langmuir troughs as described in detail elsewhere (Amado et al. 2008; Kerth et al. 2009). The two troughs are mounted on an automated movable sample stage that repeatedly shuttles between the troughs which contain the reference and the sample. Protein solutions of specific concentrations were prepared in the small trough measuring 60 × 60 × 3 mm^3^. Protein adsorption was started applying the procedure detailed in the previous section. The protein adsorption phase was considered finished, when the change of the surface pressure was less than Δ*π* = 0.2 mN m^−1^ h^−1^. Due to technical limitations of the setup and the method, stirring was not possible in the IRRAS trough. Therefore, aliquots of the Tween solution were injected at five different positions of the trough. Then, p-polarized IRRA spectra were collected with an angle of incidence of 40°, 4 cm^−1^ resolution, and 2000 scans per spectrum. Reflectance-absorbance (RA) spectra were calculated as* RA* = − log(*R*/*R*_0_) with *R*, the reflectivity of the film covered surface and *R*_0_ the reflectivity of a pure buffer surface contained in the second trough. Data analysis was done by means of ‘OPUS’ software from Bruker. Water vapour compensation was achieved with the ‘atmospheric compensation’ option of the ‘OPUS’ software and manual subtraction of water vapour spectra. Criterion for the quality of these manipulations was a horizontal line in the region of 1700–1750 cm^−1^.

### Transmission IR spectroscopy

Transmission IR spectra of HSA solutions were recorded using a “Bruker Vertex 70” FTIR spectrometer and a “Bruker Aquaspec” fluid cell with ca. 7 µm path length. As reference sodium citrate buffer (25 mM, pH 6) with sodium chloride (115 mM) was measured. Single beam spectra of the reference (*I*_0_) and the sample (*I*) spectra were recorded with 256 scans and a resolution of 4 cm^−1^. Absorbance (*A*) spectra were calculated by: *A* = − log(*I*/*I*_0_). Data analysis was done by means of ‘OPUS’ software from Bruker and compensation of residual water vapour from the sample chamber was achieved with the software’s ‘atmospheric compensation’ function.

## Results and discussion

### Displacement experiments

**Fig. 2:**
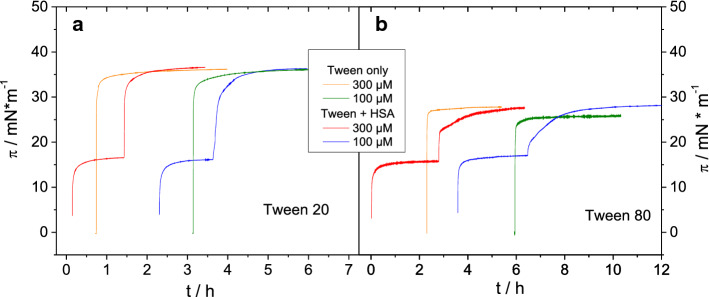
Surface pressure vs. time. Initial adsorption of protein film from HSA (5 mg ml^−1^) solutions followed by injection of Tween 20 (**a**) and Tween 80 (**b**). Injection of Tweens into pure buffer (25 mM citrate, 115 mM NaCl, pH 6, 20 °C) is shown for comparison

We first were interested in the question whether a protein film formed at the air water interface of a protein solution could be displaced by the addition of a surfactant, which is also surface active. All injection experiments therefore began with the formation of a protein film by adsorption of HSA to the air–water interface. Albumin films formed after several hours, reaching surface pressures (*π*) of ~ 16–17 mN m^−1^ (Fig. 2). Subsequently, Tween solutions were injected leading to a further increase in *π* (Fig. 2) indicating the formation of a mixed film or the displacement of the protein film by the surfactant. The surface pressure increase after injection underneath the HSA film is faster for Tween 20 than for Tween 80 (Fig. 2). Injection of Tween 20 led to a constant surface pressure after ~ 2 h whilst in case of Tween 80 it took more than 4 h to reach a constant state. In the case of Tween 80, the kinetics of surface pressure change is clearly biphasic. Tween 20 is more surface active and thus reaches higher surface pressure values up to 35 mN m^−1^ compared to Tween 80, with *π* = 25–28 mN m^−1^ as seen in the reference curves in Fig. 2 where Tween was injected into pure buffer sub-phase. However, similar adsorption kinetics were observed for both surfactants, indicating that in this case probably diffusion to the surface is the rate limiting step. Similar results were reported before by others (Samanta and Ghosh 2011). In the experiment depicted in Fig. 2, the final surface pressures reached after Tween injection underneath the HSA films are similar compared to the surface pressure reached by the surfactant alone. It is noted that also on a premixed solution, that contained both albumin and Tween 20 at the same concentrations, adsorption occurred to result in a similar surface pressure (Supporting Information: Figure S2). No significant differences were observed for injection of Tween solutions with concentrations of 100 or 300 μM, respectively. This is due to the fact that both concentrations are above the critical micellar concentration (cmc) (Samanta and Ghosh 2011; Hait and Moulik 2001; Carnero Ruiz et al. 2003).

The different kinetics for the two Tweens when interacting with the HSA film are a consequence of the differences in the molecular area at the interface of the two surfactants, as the final surfactant concentrations in the trough are above their critical micellar concentrations (cmc). Due to its longer and unsaturated chains, a Tween 80 molecule requires a higher molecular area at the interface than Tween 20 under the same conditions (Fig. 1) (Samanta and Ghosh 2011; Cross et al. 2004). This increases the energy barrier of activation for controlled adsorption and thereby slows down the adsorption rate (MacRitchie 1990). The higher area per molecule of Tween 80 at the interface also causes a lower film density (Samanta and Ghosh 2011; Cross et al. 2004), resulting in a lower final surface pressure. A difference in the progress of the protein film displacement or in the final state of the surface films is likely, due to this different adsorption behaviour of the surfactants and is studied further below.

### Infrared reflection absorption spectroscopy (IRRAS)

#### Pure HSA films

**Fig. 3:**
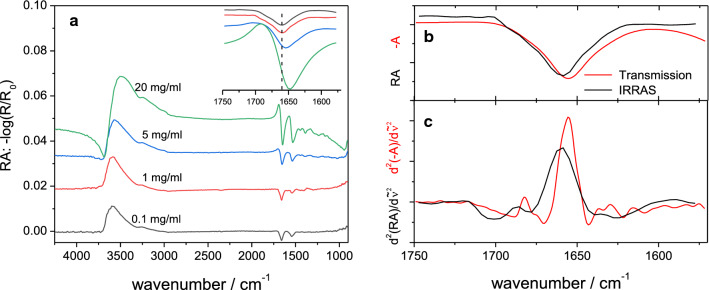
**a** IRRA spectra of adsorbed HSA films and comparison of the amide I bands in the inset, **b** comparison of transmission FTIR and IRRA spectra and **c** their second derivatives. In **b** and **c**, the IRRA and the transmission IR spectra were recorded at 1 and 5 mg ml^−1^ HSA concentration, respectively. Spectra in **b** and **c** are overlaid and scaled for the purpose of comparability. All spectra were recorded with HSA solutions in buffer: 25 mM citrate, 115 mM NaCl, pH 6, 20 °C

Representative IRRA spectra of the adsorbed albumin films show an increase of the negative amide I (~ 1650 cm^−1^) and II (~ 1550 cm^−1^) peaks due to a higher surface coverage upon higher protein concentration (Fig. 3a). The presence of these bands proves the presence of protein at the surface. The amide I maxima shifted slightly from 1659 cm^−1^ at a sub-phase concentration of 0.1 mg ml^−1^ to 1647 cm^−1^ at 20 mg ml^−1^ (Fig. 3a and Table 1). A lower shift is observed at the amide II band. Furthermore, a positive feature at the high frequency side of the amide I around 1690–1700 cm^−1^ increases with concentration. These changes are not necessarily a sign of changes in the protein secondary structure and can be explained by the optical properties of protein films at interfaces (Meinders et al. 2001), though a change of protein conformation is also possible and sometimes observed (Martin et al. 2003; Meinders et al. 2002). The shape of the amide I bands at sub-phase concentrations of 0.1 and 1 mg ml^−1^ are very similar (Fig. 3a). However, a direct comparison of the IRRAS amide bands of the higher concentrations was not possible, because the positive features, the intensity changes and the band shifts significantly hamper this comparison. For comparison with the bulk structure of HSA, a transmission IR spectrum of HSA solution was measured (Fig. 3b, c). This spectrum features a main amide I peak centred at 1655 cm^−1^, indicating a mainly α-helical secondary structure as reported before (Li et al. 2006; Curry et al. 1998). The overlay with the IRRA spectrum at 1 mg ml^−1^ albumin concentration shows, next to a minor peak shift, no major change in the peak shape. This comparison indicates that upon adsorption from concentrations ≤ 1 mg ml^−1^ no major secondary structure changes occur in HSA upon adsorption to the air–water interface. Similar conclusions for serum albumins have been drawn earlier from neutron specular reflection measurements and surface tension measurements (Lu et al. 1999; Makievski et al. 1998). The secondary structure of drug proteins at the air–water interface has been studied recently by IRRAS. For different monoclonal antibodies adsorbed at the air–water interface, the IRRAS experiments showed a clear presence of the proteins at the interphase also with no relevant change in the protein secondary structure (Koepf et al. 2017,2018a, b, c).

**Table 1:** Wavenumber of amide band (protein) maxima found in IRRAS experiments

*c*_0,HSA_/mg ml^−1^	Maximum before Tween 20 (polysorbate 20) injection		Maximum after Tween 20 (polysorbate 20) injection	
	Amide I/cm^−1^	Amide II/cm^−1^	Amide I/cm^−1^	Amide II/cm^−1^
0.1	1659	1539	–	–
1	1659	1539	–	–
5	1655	1535	1647	1524
20	1647	1531	1643	1527

A positive OH stretching band centred around 3500 cm^−1^ is observed because water is replaced by the protein film compared to the reference surface. With higher albumin concentration, the maximum of the OH stretching vibration band is red shifted, increases in intensity and a negative component appears at 3700 cm^−1^. These changes and the positive band around 1700 cm^−1^ together with the increasing negative intensity of the amide I and II bands are typical features of spectra of adsorbed protein films at high protein concentration in the sub-phase. (Meinders et al. 2001,2002; Martin et al. 2003; Kudryashova et al. 2003; Blume and Kerth 2013). Simulated spectra show this behaviour under the assumption of a one layer model with increasing layer thickness and decreasing ratio of surface to sub-phase concentration (Meinders et al. 2001). The described spectral changes can therefore be interpreted as a consequence of an increased protein layer thickness. Neutron reflection measurement implied layer thickness increase of serum albumins from ca. 3–4 nm at 0.1 mg ml^−1^ to 4–7 nm at 1 mg ml^−1^ (Lu et al. 1999) which agrees with our IRRAS results.

#### Displacement experiments of HSA films with Tween 20

For a further characterization of the HSA film properties after adsorption, we conducted displacement experiments with Tween 20 using the IRRAS setup and a series of protein concentrations (*c*_0 HSA_: 0.1; 1; 5; 20 mg ml^−1^).

**Fig. 4:**
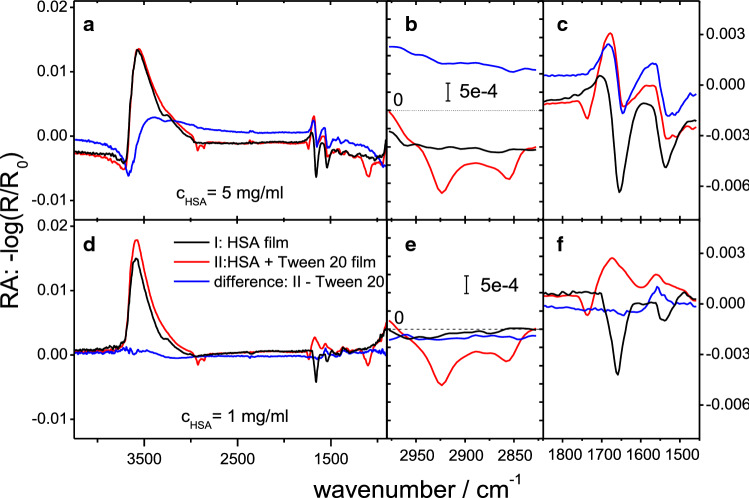
Examples of IRRA spectra during film displacement experiments at two different protein concentrations in the sub-phase. Pure HSA films, mixed films 8 h after Tween 20 (Polysorbate 20) injection (*c*_End_ = 100 µM); and difference spectra: RA(mixed film) − RA(Tween 20) are compared. The panels show full spectra (**a**, **d**), region of CH_2_ stretching bands (**b**, **e**) and amide region (**c**, **f**). The colour code applies to (**a**–**f**). Sub-phase: 25 mM citrate, 115 mM NaCl, pH 6, 20 °C

After protein adsorption and equilibration, Tween 20 was injected into the sub-phase. In contrast to the displacement experiments measuring only the pressure or using BAM, it was not possible to stir the sub-phase in the Langmuir trough used in IRRAS experiments. For a sufficiently homogeneous distribution of the surfactant, five injections at different positions were performed (for a detailed discussion on the influence of stirring see Supporting Information). Thus the time course of the displacement may be different compared to the other experiments and therefore only spectra in the final steady state (i.e. at least 8 h after injection) are discussed here. Injection of Tween 20 (final trough concentration: 100 µM), under the adsorbed protein layers, caused drastic changes in the IRRA spectra. The antisymmetric and symmetric CH_2_ stretching vibrational bands of the surfactant fatty acyl chains appear at 2923 cm^−1^ and 2855 cm^−1^, respectively (Fig. 4, middle panel). Also, the C=O stretching vibrational band of the ester groups appear at 1736 cm^−1^ indicating the presence of the surfactant at the interface.

The amide bands drop in intensity due to a partial or complete displacement of the protein from the interface, depending on the protein concentration. The region below 1700 cm^−1^ shows positive and negative features, comparable to spectra of a pure Tween 20 film (compare Figure S3, Supporting Information) and the presence of amide bands after Tween injection and cannot be easily discerned. Therefore, difference spectra were calculated by subtracting spectra of adsorbed Tween 20 films of the same sub-phase concentration from the spectra of the mixed films. The difference spectra for films formed with 1 mg ml^−1^ protein concentration show almost no amide band intensities in contrast to the spectra taken for solutions with 5 mg ml^−1^ HSA (Fig. 4). It should also be noted that these difference spectra show neither C=O nor CH_2_ stretching vibrational bands, which implies a similar surface concentration of the pure surfactant film compared to the mixed film. Whether the protein is still present in the end state of the displacement experiments can be determined by the presence of amide I and II peaks in the difference spectra (Table 1). At protein concentrations of 5 mg ml^−1^ or higher, the injection of Tween 20 with a final concentration of 100 μM is not sufficient for a complete displacement of the protein film. However, at lower HSA concentration of 1 mg ml^−1^, no amide bands were seen after Tween 20 injection and thus no or only very little protein remained at the interface. These results show, that the initial sub-phase concentration of the protein has a strong influence on the properties of the final state of the adsorbed film. If the HSA concentration in the sub-phase is higher, Tween 20 is not able to completely displace HSA from the interface.

### Brewster angle microscopy (BAM)

#### Displacement experiments with Tween 20 and Tween 80

**Fig. 5:**
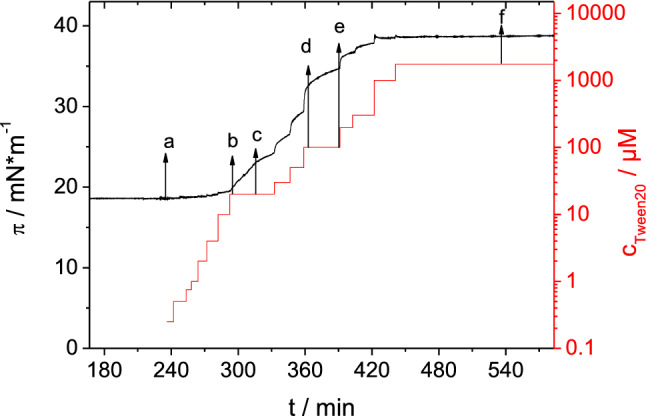
Surface pressure and surfactant concentration versus time after multiple injections of Tween 20 under an adsorbed HSA film (*c*_0_ = 20 mg ml^−1^). BAM images captured at time points a–f are displayed in Fig. 6

**Fig. 6:**
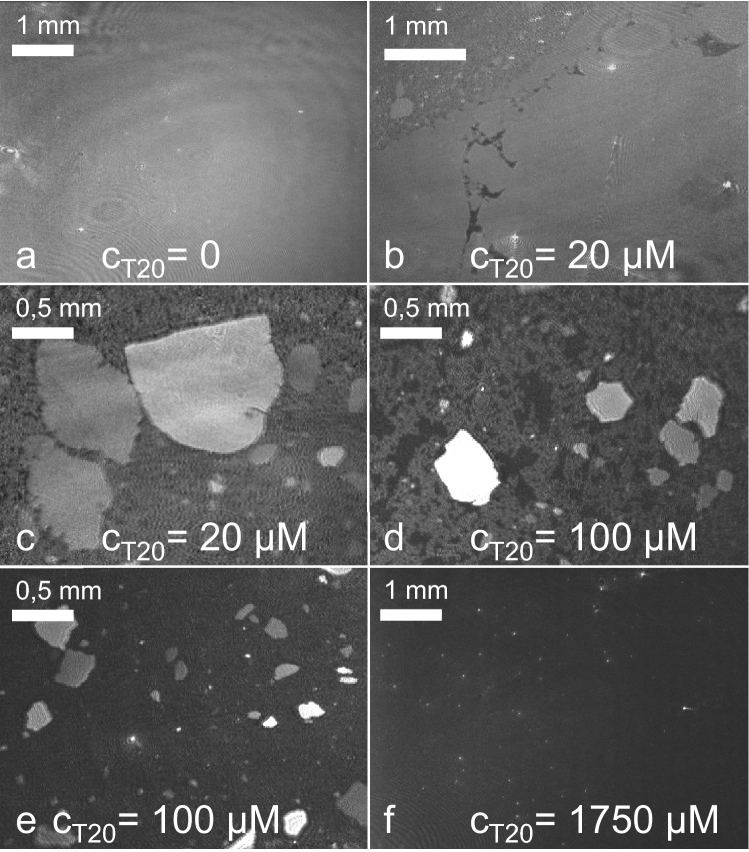
Representative BAM images of displacement of an adsorbed HSA film (*c*_0_ = 20 mg ml^−1^) by Tween 20 in a multiple injection experiment. Time points of captured images are indicated by arrows in Fig. 5. Sub-phase: 25 mM citrate, 115 mM NaCl, pH 6, at 20 °C

By means of Brewster angle microscopy, the adsorption and displacement of HSA can be observed in-situ. First, experiments were conducted with the aim of a qualitative description of the displacement process by injecting several increasing amounts of surfactant (trough concentrations: 0.25 μM–2 mM) under pre-adsorbed protein layers at a high HSA concentration of 20 mg ml^−1^ in the sub-phase (Fig. 5). This allowed the observation of the displacement under accelerated conditions, if it is assumed that the progress of this process only depends on the surface pressure and molecular effects like binding can be neglected at the interface. The sub-phase concentration of the surfactant then only influences the speed by which the surface pressure increases. Manifold changes in the film morphologies can be observed during these experiments as shown in Fig. 6.

At a sub-phase concentration of 20 mg ml^−1^ HSA films are formed with a final surface pressure of *π* = 18.5 mN m^−1^. Prior to any surfactant injection, the film appears mostly homogenous, except for small bright spots originating from smaller impurities (Fig. 6a). All protein films show higher reflectivities, i.e. brighter images compared to an empty water surface (image not shown). In all BAM experiments the sub-phase was stirred carefully between image capturing. Stirring did not cause an observable movement of the bright spots, the intact protein film seemed relatively solid and showed no lateral flow.

The injection of Tween 20 up to a concentration of 10 µM does neither influence the surface pressure (Fig. 5) nor does the BAM image of the surface change. Only when the concentration is raised to 20 µM, the surface pressure starts rising and the film morphology changes drastically (Fig. 6b). The images show three different areas of reflectivity: (1) bright areas as before on the pure HSA film, (2) dark gray (Fig. 6b, left top corner) and (3) very dark almost black areas (Fig. 6b, middle). Assuming that a possible bare water interface is immediately covered by the fast adsorbing surfactant molecules, darker areas can be assigned to surfactant rich zones, due to their lower refractive index, which leads to lower reflectivity (Mackie et al. 2011). In the black areas (3) lateral motion is not discernible, these zones seem to be cracks in the protein network and might be caused by mechanical stress as for instance the puncture of the injection needle and stirring. However, it is evident that these fractures appear only when surfactant is present and mechanical stress occurred (cf. Supporting Information Sects. 2.2–2.3). It has been shown before that compression and mechanical stress can lead to loss of protein material from the interface, which, when no other species are present, is followed by re-adsorption of protein (Koepf et al. 2017, 2018a). The surfactant, however, adsorbs faster to the interface than the protein and will rapidly cover free surface by a dense packing (Morris and Gunning 2008).

The dark gray areas (Fig. 6b) of intermediate reflectivity show a significantly different appearance. Upon stirring, distinct lateral movement and mixing can be observed in these patches. They seem to consist of loosely floating protein material. Their morphology, i.e. the lower reflectivity and the increased mobility might be explained by the existence of small surfactant domains of sizes below the lateral resolution of the BAM, i.e. in dimensions of micrometers. Also, sharply delimited, coherent islands of similar reflectivity as the initial protein film were observed, co-floating laterally in the stream of the dark gray area. The islands are able to rotate but keep their shape during all movements.

Upon further slow increase of *π* with time at constant surfactant concentration in the bulk, the observable protein film breaks up into islands with dimensions of 20–1000 µm, which float on the surface surrounded by dark gray, loose protein material (Fig. 6c). When the surfactant concentration is increased even further, the surface pressure rises and the black areas increase in size, i.e. domains of adsorbed Tween can be observed (Fig. 6d). They are irregular and variable in shape and strongly branched which is a sign for local heterogeneity in the elastic properties of the protein network (Morris and Gunning 2008).

At a Tween 20 concentration of 100 µM and a surface pressure of 34.7 mN m^−1^, only few dark grey areas of loosely packed protein remain (Fig. 6e), disappearing upon further pressure increase. The islands decrease in size over a period of ~ 2 h. The final state of the interface at a surface pressure of 38.7 mN m^−1^ still shows small, bright spots originating from the HSA film (Fig. 6f). Further increase of surfactant concentration has no influence on the surface pressure because the cmc of Tween 20 is exceeded. The maximal achieved surface pressure is not sufficient to displace the remaining parts of the protein network.

We repeated these BAM experiments with different protein concentrations. In all cases, similar patterns of the displacement process were observed. These showed similarities to the displacement of other protein films studied before, for instance, spread layers of β-lactoglobulin and β-casein (Mackie et al. 2001; Morris and Gunning 2008). For a better comparison of the experimental results, we defined four phases for the displacement process.

Phase 0: Injection of surfactant leads to no significant change in surface pressure or film morphology.

Phase I: Formation of surfactant domains and breaking of protein film.

Phase II: Shrinking of small (µm-range) protein domains. This phase ends with the critical surface pressure *π*_c_.

Phase III: Shrinking of solid protein islands (100 µm-range).

It is important to note that these phases are of descriptive value, only. The defined end pressures do not necessarily define the starting pressure of the following phase. For instance it can be assumed that phase II, the shrinking of the small protein domains, already commences before the breaking of the protein film in phase I is finished. The assigned phases after analysis of the BAM images are summarized in Table 2.

**Table 2:** Phases of the displacement of HSA films by Tween 20 and 80

*c*_protein_/mg ml^−1^	Tween 20			Tween 80	
	Spread HSA-film^a^	1	20	1	20
*π*_0,protein_/mN m^−1^	17.7	17.3	18.7	18.1	19.4
Phase 0					
*π*_END_/mN m^−1^	–	17.6	19.5	19.1	19.9
Phase I					
*π*_END_/mN m^−1^	–	19.0	21.4	20.2	21.7
Phase II					
*π*_C_/mN m^−1^	21.6	22.6	36.5	23.5	~ 34^b^
Phase III					
*π*_END_/mN m^−1^	–	37.1	38.8	35.6	35.2
Protein in final state	No	No	Yes	No	Yes
Islands of different reflectivity	No	No	Yes	No	Yes

^a^A spread film of HSA was compressed to a starting pressure of 17.7 mN m^−1^ and surfactant was injected afterwards into the sub-phase. Only Phase II of the displacement process was observed in this experiment

^b^Value obtained from extrapolation of protein surface coverage to 0%

Reason for the appearance of an initial phase 0, without observable effects, could be binding of surfactant to HSA molecules in the sub-phase. ITC experiments showed that Tween 20 and 80 bind to HSA in the volume phase with molar ratio of 1:1 or higher and binding constants of ~ 1000–1600 M^−1^ (Garidel et al. 2009). An HSA concentration of 1 mg ml^−1^ is equivalent to a molar concentration of ~ 15 µM. Thus the HSA concentration is much higher than the surfactant concentration. The initially injected surfactant then first binds to HSA in the sub-phase and therefore loses its surface activity. The protein is in excess until phase 0 ends and Tween starts to adsorb onto the surface. Hence, the surface activity of the surfactant is only slightly reduced due to presence of the protein in the sub-phase. In case of a spread HSA layer no phase 0 was observed (Table 2) due to a complete lack of protein in the sub-phase, which further confirms this interpretation.

#### Protein islands

**Fig. 7:**
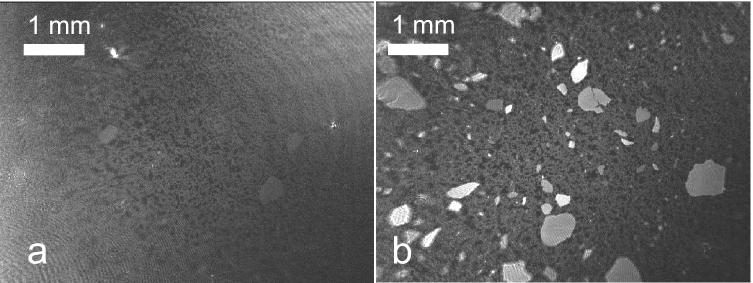
Typical images for phase II in a displacement experiment using Tween 20; **a** HSA (*c*_0_ = 1 mg ml^−1^; *π* = 20.2 mN m^−1^; *c*_Tween20_ = 5 μM); **b** (*c*_0_ = 20 mg ml^−1^; *π* = 28.6 mN m^−1^; *c*_Tween20_ = 50 μM). Sub-phase: 25 mM citrate, 115 mM NaCl, pH 6, at 20 °C

Formation and shrinking of protein islands are processes observed throughout the whole displacement experiments for all used protein concentrations and surfactant types except for a spread HSA layer. In phase I the breaking of the protein film due to shear forces upon stirring can be observed. The formation of protein islands requires both, presence of surfactant and the shear forces caused by the stirring as comparison experiments showed (Supporting Information Sect. 2.2). After the complete breakup in phase II, these islands float freely on the surface and begin to shrink. As can be seen from Table 2 and Fig. 7, which shows typical captured images of the displacement of HSA by Tween 20 in phase II, the appearance of these protein islands depends on the initial protein concentration (Fig. 7, Table 2). In case of 1 mg ml^−1^ protein concentration, few protein islands of similar reflectivity are observed (Fig. 7a). While in case of a concentration of 20 mg ml^−1^ HSA, significantly more islands as well as islands of different reflectivity can be observed (Fig. 7b). In contrast to these observations, there seems to be no significant difference on the appearance of the small dark gray protein domains on this imaging scale. However, it has to be noted that the Tween 20 concentration needed to reach phase II depends on the HSA concentration. For an HSA concentration of 20 mg ml^−1^, the Tween 20 concentration needed to reach phase II is 50 μM, i.e. a factor of 10 higher than at a concentration of HSA of 1 mg ml^−1^.

The different propensity of the albumin layers to form protein islands, depending on the protein concentration can be interpreted as a sign of differing gelation tendency. The formation of solid-like protein islands of the observed size dimensions was also reported for the displacement of adsorbed β-casein by the ionic surfactant sodium dodecylsulfate (SDS) (Bantchev and Schwartz 2004). In particular, dilute protein solutions (~ 0.05 mg ml^−1^) led to adsorbed films that broke after aging (48 h) into islands when SDS was added to the sub-phase. The films were stabilized by a strong protein network with gel character. For fresh films (2 h) no such behaviour was observed. In an earlier study (Bantchev and Schwartz 2003), it was shown that adsorption from higher concentrated protein solutions can also lead to a gelation processes in the adsorbed films after ca. 3–4 h, which is comparable to the ages of the HSA films studied here. It is also stated that these gelation processes can take much more time than the actual adsorption process. Thus the increased number of protein islands observed here for HSA indicates an increased gelation of the studied protein films.

An orogenic displacement process as proposed by Mackie et al. (1999) requires an initial adsorption of surfactant molecules into holes or defects of the protein network. This process is obviously locally hindered in the regions where protein islands exist. Recently, protein film heterogeneities, in size dimensions comparable to the islands found here, were found by BAM in intact films formed from immunoglobulins G (Koepf et al. 2017, 2018a, b). The heterogeneity of the initially formed protein film causes local differences in the initial surfactant adsorption. As shown by our IRRAS measurements, the average HSA film thickness is increased at higher protein concentrations. It can be expected that this leads to the existence of less accessible holes and defects. Thus, an increased portion of the film resists the initial surfactant adsorption, which causes the observed increased formation of islands. In the further progress of the displacement, the islands also do not seem to dissolve but rather smear out on the edges and shrink. This is best observed in phase III after the complete displacement of the loose protein material. This process can be observed until further Tween injection causes no further *π* increase which we defined as *π*_END_ of phase III. At this point the end state of the system is reached and in case of higher protein sub-phase concentrations, remaining protein islands can be found (see Table 2). In this point, the results of the BAM image analysis agree with the results obtained from the IRRAS measurements for the final film state after displacement. At HSA concentrations ≥ 5 mg ml^−1^, the surfactant could not fully displace these parts of the heterogeneous protein film and residual amide bands were seen (see Fig. 3). We conclude that an orogenic mechanism is prevented in these areas due to a to strong protein network in combination with increased protein layer thickness which hinders surfactant co-adsorption.

An interesting detail is the existence of protein islands of different brightness, i.e. different reflectivity, during the displacement process (Fig. 7b), an observation which was only seen in case of films formed from higher concentrated HSA solutions (Table 2). The differences in brightness remained similar irrespective whether the islands rotated or moved relative to the image frame, or moved relative to each other. This excludes anisotropic refractive indices in the films and uneven illumination as possible reasons. A more likely reason for the different reflectivities is a difference in layer thickness. Small protein domains during the displacement processes showed such thickness differences (Mackie et al. 1999) and local thickness differences were found also within intact films of immunoglobulin G using BAM (Koepf et al. 2017, 2018a). As shown in these studies, an increase in surface pressure led to significant increases in protein layer thicknesses. The typical layer thickness of adsorbed albumin films was found to be ~ 3 nm, which implies a monolayer with flat orientation of the molecules on the surface (Hansen and Myrvold 1995; Lu et al. 1999). Formation of bilayers and conformational changes were shown to increase the thickness up to 4–7 nm. It must be noted that different reflectivities in the BAM may also be caused by an increased effective refractive index of the protein layer, i.e. by an increased packing density which cannot be excluded by our data. However, as discussed above, the increased layer thickness due to surface pressure increase of protein films is well documented in the literature and thus appears likely. The increased surface pressure upon displacement by Tween leads to a compression of the protein film. Therefore, the appearance of protein islands with different reflectivities shows that the heterogeneous protein network shows also locally different responses manifesting in protein islands with different film thicknesses or packing densities.

#### Small protein domains

The small, grey, floating protein domains are found in all displacement experiments with HSA in the intermediate surface pressure regime (see Table 2). They appear with the first break-up of the rigid protein film and disappear at a distinct surface pressure *π*_c_ that coincides with the collapse of the protein network proposed by Mackie et al. (2001, 1999). The values of this critical surface pressure are independent on the type of surfactant (Table 2), as it is expected for an orogenic displacement (Mackie et al. 1999). Higher HSA concentration leads to a stronger network structure, resulting in an increased critical surface pressure. In case of the multiple injections of Tween 80 under an adsorbed film of HSA with a sub-phase concentration of 20 mg ml^−1^, the surface pressure after the final injection increased very slowly. Because of water evaporation the BAM image quality deteriorated during this time. The critical surface pressure in this case was determined by extrapolation of the protein surface coverage to 0 (Table 2) and is an approximation. Still it is clearly much higher than the corresponding π_c_ at lower protein concentrations.

#### Quantification of domain displacement from BAM images

**Fig. 8:**
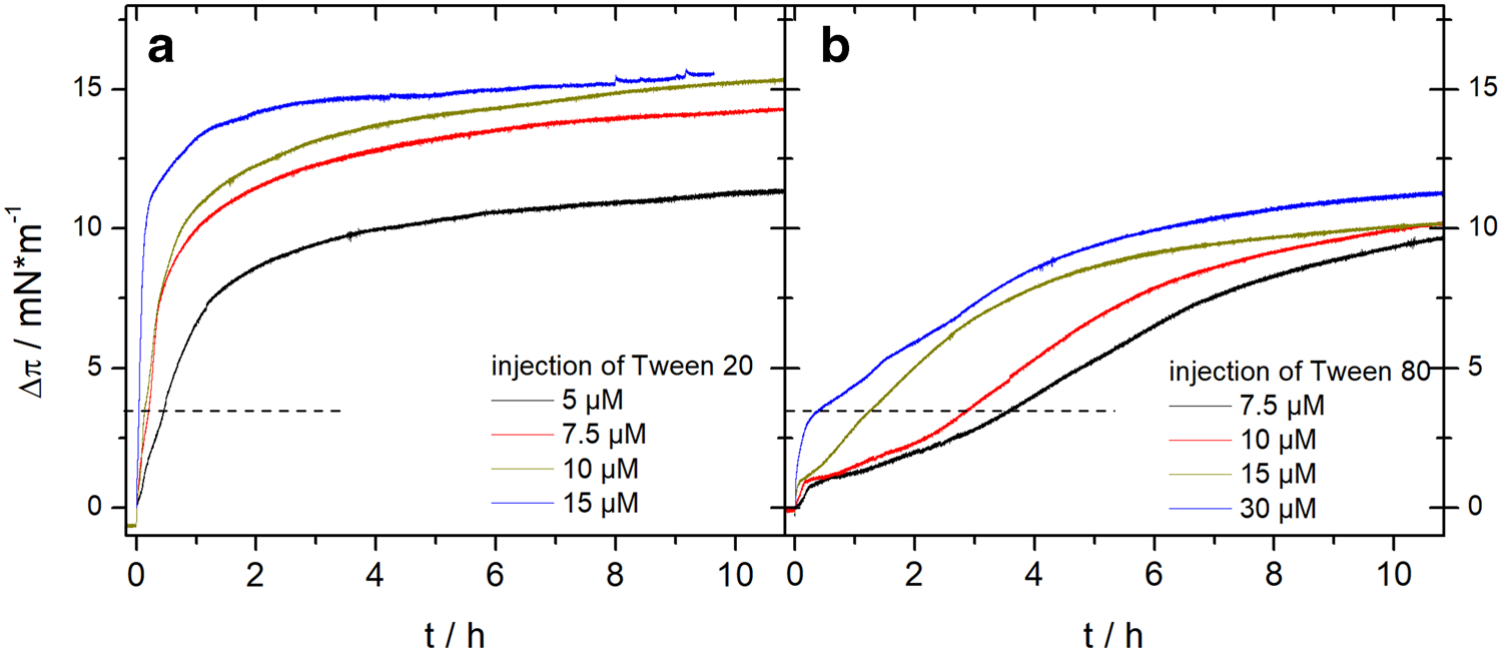
Change of surface pressure Δ*π* with time after single injection of Tween 20 (**a**) and Tween 80 (**b**) under an adsorbed protein film of an HSA solution (1 mg ml^−1^). Moment of injection: *t*_0_ = 0; Initial surface pressure of adsorbed HSA film: *π*_0_ ~ 18 mN m^−1^. Black dashed line: critical surface pressure as shown in Fig. 9. Sub-phase: 25 mM citrate, 115 mM NaCl, pH 6, at 20 °C

In order to quantify the displacement of the small protein domains from the interface depending on time or surface pressure, we performed a different set of experiments, where single injections of surfactants lead to different final surfactant concentrations in the sub-phase. In contrast to the experiments discussed above, the final surfactant concentrations were always below the cmc to slow down the displacement process to be able to obtain sufficient BAM images. For these experiments the HSA concentration was always 1 mg ml^−1^. For the quantification of the BAM images histograms were analysed as described in the Methods section and schematically shown in Figure S1 of the Supplementary Information. The surface pressure changes (Fig. 8) observed after surfactant injection follow the expected trends seen before (see Fig. 2). The adsorption process of Tween 20 compared to Tween 80 was much faster and also led to a higher final surface pressure (Fig. 8).

**Fig. 9:**
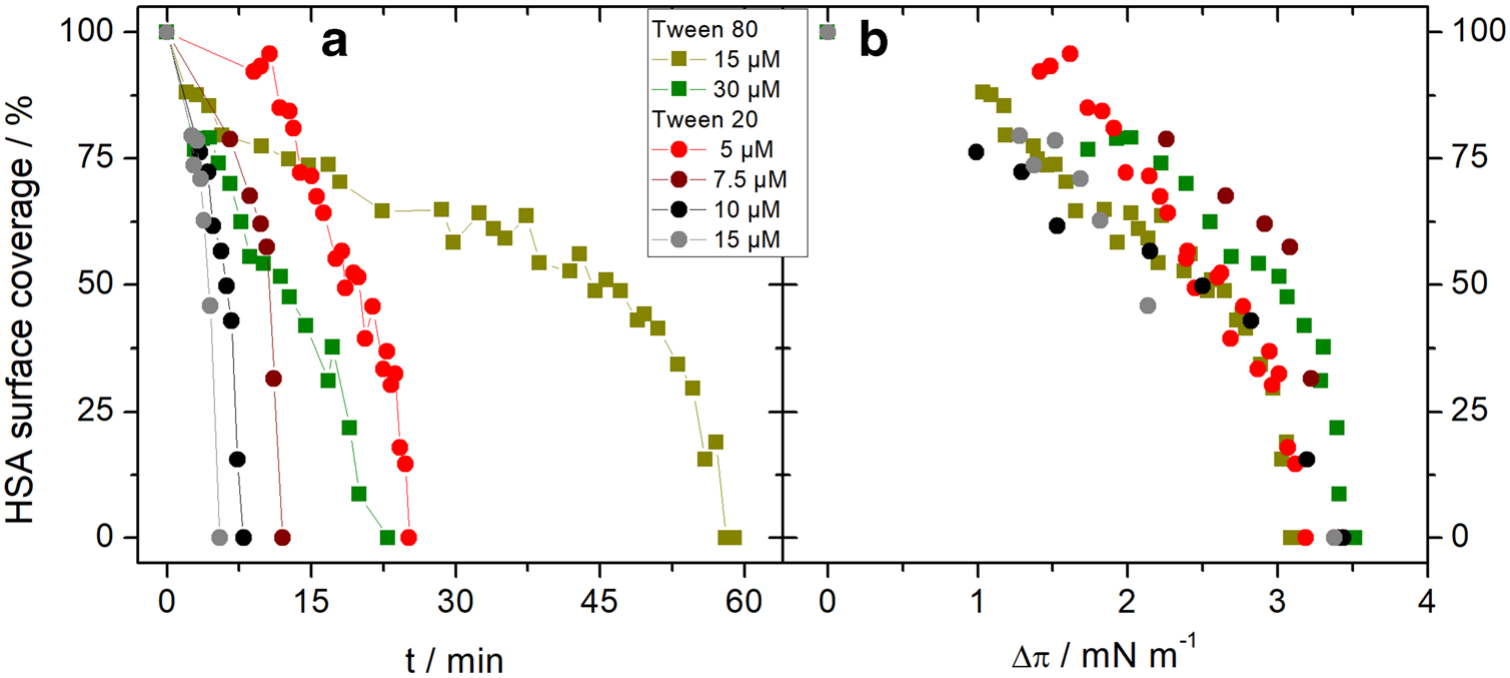
Protein surface coverage after single injection of Tween (polysorbate) 20 or Tween 80 under an adsorbed HSA film on a solution with *c* = 1 mg ml^−1^. Surface pressure of adsorbed HSA film: *π*_0_ ~ 18 mN m^−1^. Moment of injection: *t*_0_ = 0; Data are plotted against time (**a**) or change in surface pressure (**b**). Sub-phase: 25 mM citrate, 115 mM NaCl, pH 6, at 20 °C

The behaviour of the protein surface coverage in time (Fig. 9a) shows similar tendencies as the surface pressure development (Fig. 8). The protein is displaced faster with increasing surfactant concentration and when Tween 20 is used instead of Tween 80. In contrast, there is no dependence on the nature of the surfactant or on the concentration when the HSA surface coverage is plotted against the change in surface pressure (Fig. 9b). In all performed experiments, independent of time and surfactant type, the small protein domains were totally displaced from the interface after a surface pressure change of Δ*π* ~ 3.5 mN m^−1^. With the average starting pressure of 18 mN m^−1^ this leads to a critical pressure of ~ 21.5 mN m^−1^ which is in good agreement with the critical pressure found for *c*_0 HSA_ = 1 mg ml^−1^ in the multiple injection experiments (Table 2).

These results obtained for the behaviour of the small protein domains are in agreement with an orogenic displacement mechanism. The increase of surface pressure compresses the protein network which then fails and gets displaced at a critical surface pressure *π*_c_. As the structural properties of the protein networks are expected to be similar in the different experiments, the way this critical pressure is reached (“fast” adsorbing Tween 20 or “slow” adsorbing Tween 80) does not influence mechanics of the displacement process.

After the critical pressure is reached (black dashed lines in Fig. 8), the surface pressure increases further and no significant change in adsorption velocity is observable for either of the surfactants (Fig. 8). Therefore, the displacement process has no noticeable influence on the kinetics of the surfactant adsorption.

**Fig. 10:**
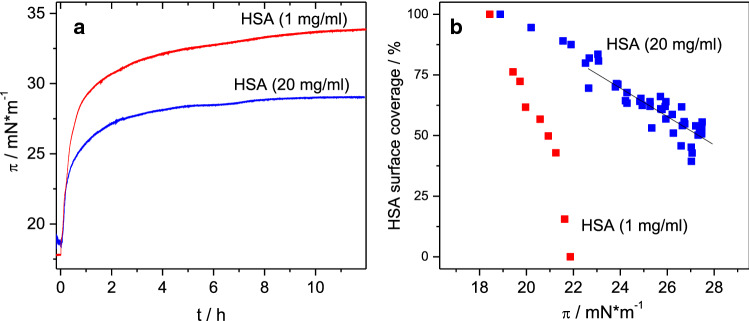
Surface pressure (**a**) and protein surface coverage (**b**) after single injection of Tweens 20 (Polysorbate 20) under an adsorbed protein film on HSA solution. Final concentration of Tween 20 was 10 µM and 20 µM for HSA with a concentration of 1 mg ml^−1^ or 20 mg ml^−1^, respectively. Moment of injection: *t*_0_ = 0; Extrapolation of the linear fit (black line) leads to a critical pressure of 35.9 mN m^−1^. Sub-phase: 25 mM citrate, 115 mM NaCl, pH 6, at 20 °C

The dependency of the critical pressure on the protein concentration, as found in the multiple injection experiments (see Table 2), was also seen using a single injection leading to a final surfactant concentration below the cmc, using 1 and 20 mg ml^−1^ albumin concentrations (see Fig. 10).

The surfactant is less able to displace the protein film in case of a higher protein concentration (Fig. 10a), because the protein surfactant binding in the sub-phase reduces the active concentration of the surfactant. Also, the completion of the displacement needs a higher surface pressure at 20 mg ml^−1^ albumin concentration and could not be achieved by a single injection reaching a concentration of 20 µM Tween 20 (Fig. 10b). Extrapolation of the linear fit (black line in Fig. 10b) to a surface coverage of 0% leads to a surface pressure of ~ 36 mN m^−1^, which is in good agreement with the critical pressure π_c_ for HSA at 20 mg ml^−1^ found in the multiple injection experiments (Table 2).

## Summary and conclusions

In this study spectroscopic and microscopic techniques were used in combination with surface pressure measurements to shed light on important details of the displacement of human serum albumin (HSA) from the air–water interface by non-ionic surfactants. The collected data of the displacement of the adsorbed HSA films by the Tweens 20 and 80 can partially be understood on the basis of an orogenic displacement mechanism as proposed earlier (Mackie et al. 1999). The final displacement thereby depends on the critical surface pressure of the adsorbed protein film. HSA formed surface films upon adsorption. With increasing HSA concentration, the ability of Tween 20 and 80 to displace these films from the interface decreased. The displacement progressed in three phases, which were distinctly different in their surface morphology as seen in the BAM images. These different phases have their origin in the heterogeneity of the initially adsorbed protein network.

BAM allowed the observation and quantification of the displacement of protein domains of µm dimensions in-situ as a function of time. The critical surface pressure *π*_c_ depended strongly on the properties of the HSA film, but was independent of the surfactant type and its concentration. Higher concentrated protein solutions resulted in films more resistant to displacement. IRRA spectroscopy showed that up to 1 mg ml^−1^ concentrations HSA did not significantly change its secondary structure upon adsorption. Furthermore, higher protein solutions led to formation of thicker films at the interface. The higher thickness might be one reason for the higher resistance of these films to displacement by the polysorbates.

A morphological peculiarity, which is not directly considered by the orogenic displacement mechanism, is the formation of protein islands that remain at the interface beyond *π*_c_. With increasing HSA concentration in the sub-phase, the protein network on the interface increases in heterogeneity, meaning that an increasing number of domains with stronger gel-like networks exist in the film. In these areas, an orogenic displacement is apparently hindered by prevention of the initial surfactant adsorption step. This resulted in island formation, and the protein remained as coherent islands at the interface in the final state. Protein islands of different reflectivity were observed, possibly caused by different layer thicknesses.

After addition of Tween 20 as well as Tween 80, similar HSA film morphologies and the same critical pressure *π*_c_ during displacement were observed. The difference in chemical structure between the two surfactants obviously does not influence the displacement mechanism. Differences in the displacement process originate from their different adsorption properties caused by the different acyl chains. The velocity of the adsorption of both Tweens, and thus the speed of the protein displacement, increased with rising surfactant concentration. Tween 80 reached significantly lower surface pressures than Tween 20, which resulted in an incomplete displacement of the observed small protein domains.

As shown in this study, the orogenic displacement mechanism is generalizable towards systems that are relevant for parenteral drug formulations. It shows that especially the protein concentration may be an important factor influencing the properties of the formed films in such mixed protein-surfactant systems. Thus further research will need to investigate its applicability to other proteins used in such formulations.

## Electronic supplementary material

Below is the link to the electronic supplementary material.

Supplementary file1 (DOCX 3824 kb)

## References

[CR1] Amado E, Kerth A, Blume A, Kressler J (2008). Infrared reflection absorption spectroscopy coupled with brewster angle microscopy for studying interactions of amphiphilic triblock copolymers with phospholipid monolayers. Langmuir.

[CR2] Ariga K, Hill JP (2011). Monolayers at air–water interfaces: from origins-of-life to nanotechnology. Chem Rec.

[CR3] Bantchev GB, Schwartz DK (2003). Surface shear rheology of β-casein layers at the air/solution interface: formation of a two-dimensional physical gel. Langmuir.

[CR4] Bantchev GB, Schwartz DK (2004). Structure of β-casein layers at the air/solution interface: atomic force microscopy studies of transferred layers. Langmuir.

[CR5] Blume A, Kerth A (2013). Peptide and protein binding to lipid monolayers studied by FT-IRRA spectroscopy. Biochim Biophys Acta.

[CR6] Carnero Ruiz C, Molina-Bolívar J, Aguiar J, MacIsaac G, Moroze S, Palepu R (2003). Effect of ethylene glycol on the thermodynamic and micellar properties of Tween 20. Coll Polym Sci.

[CR7] Carpenter JF, Manning MC (2002). Rational design of stable protein formulations. Theory and practice.

[CR9] Cross GH, Reeves A, Brand S, Swann MJ, Peel LL, Freeman NJ, Lu JR (2004). The metrics of surface adsorbed small molecules on the young's fringe dual-slab waveguide interferometer. J Phys D Appl Phys.

[CR10] Curry S, Mandelkow H, Brick P, Franks N (1998). Crystal structure of human serum albumin complexed with fatty acid reveals an asymmetric distribution of binding sites. Nat Struct Biol.

[CR11] Den Engelsman J, Garidel P, Smulders R, Koll H, Smith B, Bassarab S, Seidl A, Hainzl O, Jiskoot W (2011). Strategies for the assessment of protein aggregates in pharmaceutical biotech product development. Pharm Res.

[CR12] Dwivedi M, Blech M, Presser I, Garidel P (2018). Polysorbate degradation in biotherapeutic formulations: identification and discussion of current root causes. Int J Pharm.

[CR13] Dwivedi M, Buske J, Haemmerling F, Blech M, Garidel P (2020). Acidic and alkaline hydrolysis of polysorbates under aqueous conditions: towards understanding polysorbate degradation in biopharmaceutical formulations. Eur J Pharm Sci.

[CR14] Falconer RJ (2019). Advances in liquid formulations of parenteral therapeutic proteins. Biotechnol Adv.

[CR15] Fernández A (2016). Physics at the biomolecular interface.

[CR16] Garidel P, Blume A (2005). Dimyristoyl-sn-glycero-3-phosphoglycerol (DMPG) monolayers: Influence of temperature, pH, ionic strength and binding of alkaline earth cations. Chem Phys Lipids.

[CR17] Garidel P, Kebbel F (2010). Protein therapeutics and aggregates characterized by photon correlation spectroscopy. BioProcess Int.

[CR18] Garidel P, Hoffmann C, Blume A (2009). A thermodynamic analysis of the binding interaction between polysorbate 20 and 80 with human serum albumins and immunoglobulins: a contribution to understand colloidal protein stabilization. Biophys Chem.

[CR19] Garidel P, Blume A, Wagner M (2015). Prediction of colloidal stability of high concentration protein formulations. Pharm Dev Technol.

[CR20] Garidel P, Kuhn AB, Schäfer LV, Karow-Zwick AR, Blech M (2017). High-concentration protein formulations: how high is high?. Eur Biophys J.

[CR21] Gervasi V, Dall Agnol R, Cullen S, McCoy T, Vucen S, Crean A (2018). Parenteral protein formulations: an overview of approved products within the European Union. Eur J Pharm Biopharm.

[CR22] Graham DE, Phillips MC (1979). Proteins at liquid interfaces: I. Kinetics of adsorption and surface denaturation. J Coll Interf Sci.

[CR23] Hait S, Moulik SP (2001). Determination of critical micelle concentration (CMC) of nonionic surfactants by donor-acceptor interaction with iodine and correlation of CMC with hydrophile-lipophile balance and other parameters of the surfactants. J Surfact Det.

[CR24] Hansen FK, Myrvold R (1995). The kinetics of albumin adsorption to the air/water interface measured by automatic axisymmetric drop shape analysis. J Coll Interf Sci.

[CR25] Hoffmann C, Blume A, Miller I, Garidel P (2009). Insights into protein-polysorbate interactions analysed by means of isothermal titration and differential scanning calorimetry. Eur Biophys J.

[CR26] Kerth A, Erbe A, Dathe M, Blume A (2004). Infrared reflection absorption spectroscopy of amphipathic model peptides at the air/water interface. Biophys J.

[CR27] Kerth A, Garidel HJ, Alexande C, Mach JP, Waelli T, Blume A, Rietschel ETh, Brandenburg K (2009). An infrared reflection-absorption spectroscopic (IRRAS) study of the interaction of lipid A and lipopolysaccharide Re with endotoxin-binding proteins. Med Chem.

[CR28] Khan TA, Mahler HC, Kishore RSK (2015). Key interactions of surfactants in therapeutic protein formulations: a review. Eur J Pharm Biopharm.

[CR29] Koepf E, Schroeder R, Brezesinski G, Friess W (2017). The film tells the story: physical–chemical characteristics of IgG at the liquid–air interface. Eur J Pharm Biopharm.

[CR30] Koepf E, Eisele S, Schroeder R, Brezesinski G, Friess W (2018). Notorious but not understood: How liquid-air interfacial stress triggers protein aggregation. Int J Pharm.

[CR31] Koepf E, Richert M, Braunschweig J, Schroeder R, Brezesinski G, Friess W (2018). Impact of formulation pH on physicochemical protein characteristics at the liquid–air interface. Int J Pharm.

[CR32] Koepf E, Schroeder R, Brezesinski G, Friess W (2018). The missing piece in the puzzle: prediction of aggregation via the protein-protein interaction parameter A2. Eur J Pharm Biopharm.

[CR33] Kudryashova EV, Meinders MBJ, Visser AJWG, van Hoek A, de Jongh HHJ (2003). Structure and dynamics of egg white ovalbumin adsorbed at the air/water interface. Eur Biophys J.

[CR34] Li Y, He W, Dong Y, Sheng F, Hu Z (2006). Human serum albumin interaction with formononetin studied using fluorescence anisotropy, FT-IR spectroscopy, and molecular modeling methods. Bioorg Med Chem.

[CR35] Lu JR, Su TJ, Penfold J (1999). Adsorption of serum albumins at the air/water interface. Langmuir.

[CR36] Mackie AR, Gunning AP, Wilde PJ, Morris VJ (1999). Orogenic displacement of protein from the air/water interface by competitive adsorption. J Coll Interf Sci.

[CR37] Mackie AR, Gunning AP, Ridout MJ, Wilde PJ, Patino JR (2001). In situ measurement of the displacement of protein films from the air/water interface by surfactant. Biomacromol.

[CR38] MacRitchie F (1990). Chemistry at interfaces.

[CR39] Makievski AV, Fainerman VB, Bree M, Wüstneck R, Krägel J, Miller R (1998). Adsorption of proteins at the liquid/air interface. J Phys Chem B.

[CR40] Martin AH, Meinders MBJ, Bos MA, Cohen Stuart MA, Van Vliet T (2003). Conformational aspects of proteins at the air/water interface studied by infrared reflection-absorption spectroscopy. Langmuir.

[CR41] McAuley WJ, Jones DS, Kett VL (2009). Characterisation of the interaction of lactate dehydrogenase with Tween-20 using isothermal titration calorimetry, interfacial rheometry and surface tension measurements. J Pharm Sci.

[CR100] Medders GR, Paesani F (2016). Dissecting the molecular structure of the air/water interface from quantum simulations of the sum-frequency generation spectrum. J Am Chem Soc.

[CR42] Meinders MBJ, De Jongh HHJ (2002). Limited conformational change of β-lactoglobulin when adsorbed at the air–water interface. Biopolymers.

[CR43] Meinders MBJ, van den Bosch GG, de Jongh HH (2001). Adsorption properties of proteins at and near the air/water interface from IRRAS spectra of protein solutions. Eur Biophys J.

[CR45] Morris VJ, Gunning AP (2008). Microscopy, microstructure and displacement of proteins from interfaces: implications for food quality and digestion. Soft Matter.

[CR46] Samanta S, Ghosh P (2011). Coalescence of bubbles and stability of foams in aqueous solutions of Tween surfactants. Chem Eng Res Des.

[CR47] Torosantucci R, Furtmann B, Elshorst B, Pfeiffer-Marek S, Hartleb T, Andres N, Bussemer T (2018). Protein-excipient interactions evaluated via nuclear magnetic resonance studies in polysorbate-based multidose protein formulations: influence on antimicrobial efficacy and potential study approach. J Pharm Sci.

[CR48] Tripp BC, Magda JJ, Andrade JD (1995). Adsorption of globular proteins at the air/water interface as measured via dynamic surface tension: concentration dependence, mass-transfer considerations, and adsorption kinetics. J Coll Interf Sci.

[CR49] Van Oss CJ, Giese RF, Docoslis A (2005). Hyperhydrophobicity of the water–air interface. J Dispers Sci Technol.

[CR50] Wang W, Roberts CJ (2010). Aggregation of therapeutic proteins.

[CR51] Woodward NC, Gunning AP, Mackie AR, Wilde PJ, Morris VJ (2009). Comparison of the orogenic displacement of sodium caseinate with the caseins from the air-water interface by nonionic surfactants. Langmuir.

