# Supplementary material for: Albumin displacement at the air–water interface by Tween (Polysorbate) surfactants
Source: Eur Biophys J. 2020 Sep 11;49(7):533–47. doi: 10.1007/s00249-020-01459-4 (PMC7666296; doi:10.1007/s00249-020-01459-4)
Supplement: Supplementary file 1 — Supplementary file1 (DOCX 3824 kb) [file 249_2020_1459_MOESM1_ESM.docx]

**Supporting Information**

Albumin Displacement at the Air/Water Interface by Tween (Polysorbate) Surfactants

Martin Rabe^1,2^, Andreas Kerth^1^, Alfred Blume^1*^, Patrick Garidel^3^

^1^Institute of Chemistry - Physical Chemistry, Martin-Luther-University Halle-Wittenberg, Halle/Saale, Germany.

^2^Present Address: Max-Planck-Institut für Eisenforschung GmbH, Max-Planck-Straße 1, 40237 Düsseldorf, Germany

^3^Boehringer Ingelheim Pharma GmbH & Co. KG, Innovation Unit, PDB, D-88397 Biberach an der Riss, Germany

*Corresponding author:

Alfred Blume

Institute of Chemistry - Physical Chemistry, Martin-Luther-University Halle-Wittenberg

von-Danckelmann-Platz 4, D-06120 Halle (Saale), Germany.

E-mail address: alfred.blume@chemie.uni-halle.de

# 1. Method: Quantitative BAM image analysis


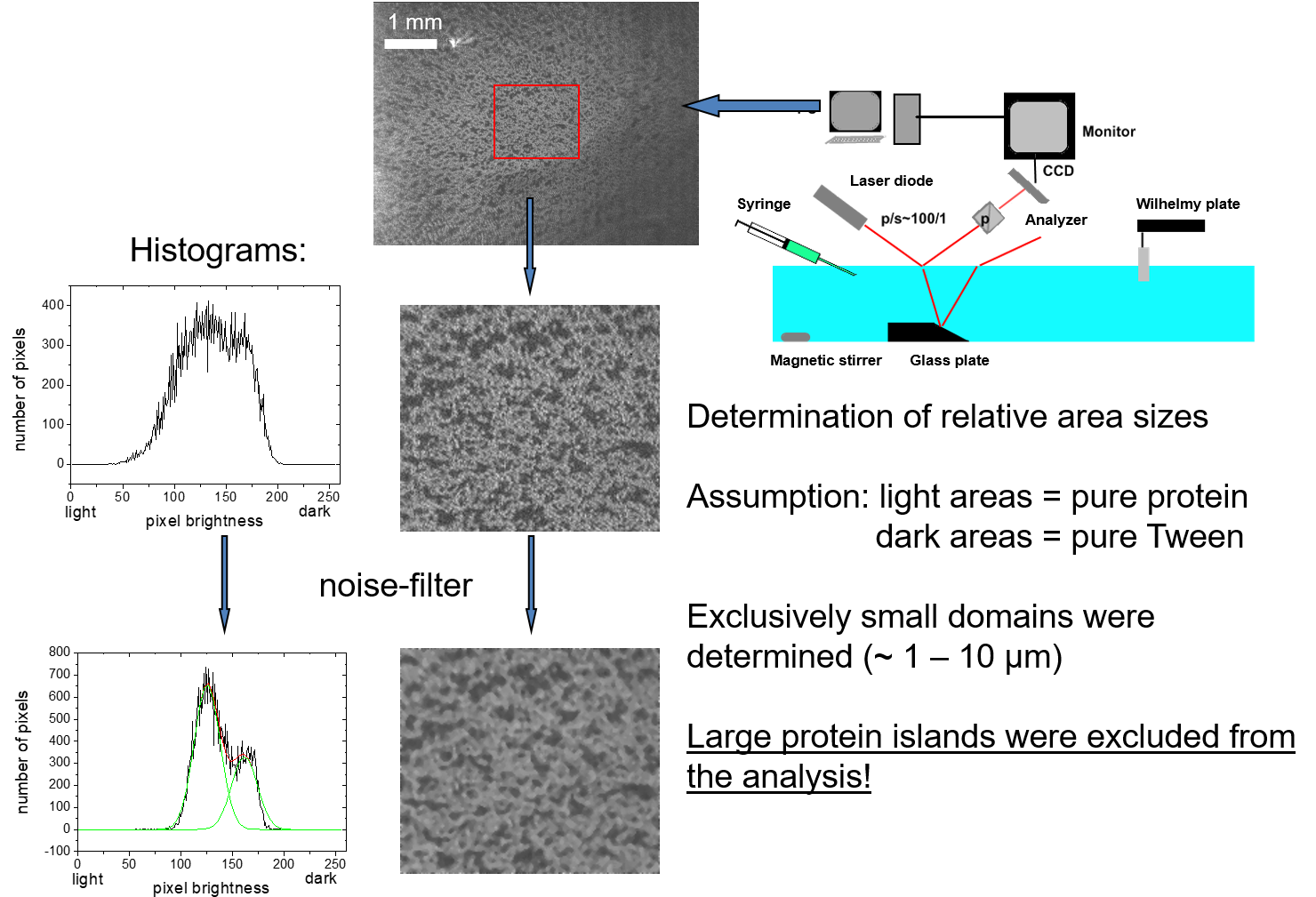


Figure S1: Scheme for the analysis of BAM images using brightness histograms.

# 2. Experimental design.

## 2.1. Order of injections.

Experiments were performed to investigate the influence of the order of the injections in the experiments. Surface pressure has been monitored after injection of a solution containing a mixture of both, Albumin (5 mg/ml) and Tween 20 (100 µM). The final pressure was found to be similar to the final pressure after injection of Tween 20 into pure buffer and under adsorbed protein film using the same concentrations (Figure S2).

Also, a reversed order of injection was tested in an IRRAS experiment (Figure S3): HSA was injected (final c = 0.1 mg ml^-1^) underneath an adsorbed Tween 20 film (c=100 µM). In this experiments no significant pressure increase and no spectral change was observed due to protein injection

Figure S2: Comparison of change in surface pressure upon adsorption of Tween 20, mixed HSA and Tween 20 solutions and subsequent adsorption of Albumin followed by Tween 20. Conditions in all experiments: HSA (5 mg/ml), Tween 20 (100 µM), Sub-phase: 25 mM citrate, 115 mM NaCl, pH 6, at 20 °C.

Figure S3: Injection of HSA under adsorbed Tween 20 film in IRRAS setup. In the surface pressure over time plot (a) the arrow indicates the moment of injection and the colors mark the moments when the IRRA spectra (b) were recorded. Conditions: HSA (0.1 mg ml^-1^), Tween 20 (100 µM), Sub-phase: 25 mM citrate, 115 mM NaCl, pH 6, at 20 °C.

## 2.2. Influence of stirring.

Initial experiments showed that a proper distribution of the detergent in the sub-phase is important for a uniform detergent adsorption over the whole surface. The Langmuir troughs used in the experiments in general are flat (5 mm height in BAM experiments, 3 mm height in IRRAS) and rectangular. The detergent cannot be distributed sufficiently by diffusion in the lateral dimensions when a single injection in a single spot at the trough bottom is performed. Thus, formation of a high local concentration should be prevented, for instance by stirring.

In the BAM experiments, discussed in the main text, gentle stirring of the sub-phase was started at the end of the protein adsorption phase. After start of the stirring, both surface pressure and surface morphology were monitored for several minutes to ensure that no major changes occurred. For the acquisition of BAM images the stirrer was put off and put back on afterwards. An example, showing the development of surface pressure and morphology for up to 20 minutes after start of the stirrer is shown in Figure S4. Before start of the stirrer, the surface has a higher reflectivity than a pure water interface and the film appears mostly homogenous, except for some brighter spots originating from small impurities (Figure S4a). After the start of the stirrer (marked by an arrow in Figure S4e), the surface pressure dropped slightly by ~0.1-0.2 mN⋅m^-1^ but remained stable after that. During the following 20 minutes, the surface morphology did not change noticeably (Figure S4b) and Tween injection was started hereafter.


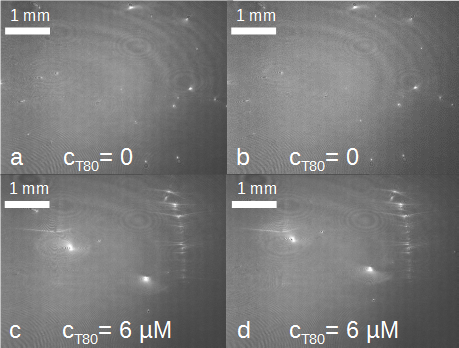


Figure S4: BAM images (a-d) of adsorbed HSA film (c_0_ = 20 mg ml^-1^) during phase 0 in multiple injection experiment. Due to injections, the image section changed between figures b and c. Moment of capturing is in the surface pressure ­­­– time plot (e). Sub-phase: 25 mM citrate, 115 mM NaCl, pH 6, at 20 °C.

Another experiment was performed to assess the effect of the stirring during the displacement (Figure S5). In displacement experiments without stirring, formation of a displacement front, i.e. a sharp boarder between a bright (protein film) and a darker (detergent rich) area (Figure Figure S5b), was observed. This observation is understood as a consequence of a local detergent adsorption to the surface, due the high local concentration. Subsequent detergent injections cause the lateral movement of the front, away from the injection position. Due to the spatial restrictions in the experimental setup, only a small section of the interface can be imaged and obtaining representative images or an overview of the state of the full surface was not possible, when strong local differences occurred as in the unstirred experiments. In the particular experiment, displayed in Figure S5, stirring of the sub-phase was started between images in Figure S5b and c. The stirring had no measurable influence on the surface pressure, yet it caused the breakup of the film into protein islands (Figure S5).

The discussed data illustrates two important findings about the influence of the stirring as performed in the BAM experiments:


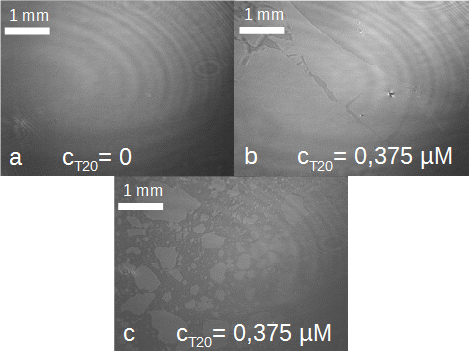


Figure S5: BAM images (a-c) of adsorbed HSA film (c_0_ = 1 mg ml^-1^) during displacement experiment. Between a and b without stirring between b and c with stirring. Moment of capturing is in the surface pressure – time plot (d). Sub-phase: 25 mM citrate, 115 mM NaCl, pH 6, at 20 °C.

I: The adsorbed albumin layer is not affected by the stirring of the sub-phase.

II: In mixed films of albumin and detergents the stirring causes the breaking of the compact protein film into smaller islands.

In IRRAS experiments stirring was not possible due to technical limitations of the setup and the method. For a better distribution in the sub-phase detergent injections were performed in 5 different positions of the trough and the repeated shuttling of the troughs in the setup caused sub-phase movements and a slow detergent distribution. However, appearance of several local spots with increased detergent concentration, directly after injection cannot be ruled out by this procedure. This most likely influences the progress of the displacement and limits the comparability of spectra obtained during the displacement process to the BAM measurements. This limitation does however not apply for spectra of the pure protein films which are directly comparable, because also in the BAM experiments stirring was started prior to detergent injection. In the main part of the paper only spectra at least 8 hours after injection are discussed when the change in the surface pressure was below 0.2 mN⋅m^-1^⋅h^-1^ and no more changes in the spectra over time were observed. The final state i.e. the presence of protein in these films was found to be dependent on the initial protein concentration, similar to the BAM experiments.

## 2.3. Influence of injections

The initial injections and the piercing of the adsorbed protein film occurring thereby did not significantly alter the surface pressure (Figure S4e). Also, during several injections in phase 0 no change in the film morphology could be observed (Figure S4c and d) and the surface pressure increased only slightly starting with the 4th injection. Note that, due to space requirements the BAM had to be moved upon injections, and thus image sections are shifted between surfactant concentration steps. A direct observation of the injection spot by BAM was not possible. Thus, the question whether local perturbations of the protein film are caused by piercing by the needle cannot be fully answered. However, as discussed in the manuscript we find it likely that film perturbations or cracks formed due to the puncture act as initial sites for detergent adsorption. An intrinsic part of an orogenic displacement is the initial adsorption of detergent into defect sites of the protein film. In our experimental design, it is very likely that the injection site acts as such defect site. On the other hand such defects maybe imposed by impurities such as dust at the interface.
